# Sex, gender, and sociodemographic factors associated with repeated prescription refills in chronic pain: insights from a prescription claims cohort study

**DOI:** 10.3389/fpain.2026.1810867

**Published:** 2026-04-22

**Authors:** Marimée Godbout-Parent, Nancy Julien, Hermine Lore Nguena Nguefack, M. Gabrielle Pagé, Line Guénette, Lucie Blais, Nancy Ménard, Sylvie Beaudoin, Anaïs Lacasse

**Affiliations:** 1 Département des Sciences de la Santé, Université du Québec en Abitibi-Témiscamingue (UQAT), Rouyn-Noranda, QC, Canada; 2 Centre de Recherche du Centre Hospitalier de l'Université de Montréal (CRCHUM), Montréal, QC, Canada; 3 Département d'anesthésiologie et de Médecine de la Douleur, Faculté de Médecine, Université de Montréal, Montréal, QC, Canada; 4 Faculté de Pharmacie, Université Laval, Québec, QC, Canada; 5 Axe Santé des Populations et Pratiques Optimales en Santé, Centre de Recherche du CHU de Québec – Université Laval, Québec, QC, Canada; 6 Faculté de Pharmacie, Université de Montréal, Montréal, QC, Canada; 7 Chaire de recherche institutionnelle en épidémiologie de la douleur chronique, Rouyn-Noranda, QC, Canada

**Keywords:** antidepresants, chronic pain, gender, medication, opioids, pharmacological treatment, sex, SGBA+

## Abstract

**Purpose:**

Chronic pain (CP) disproportionately affects women and gender-diverse individuals, raising questions about how sociodemographic factors influence medication use. Yet, the interplay between sex, gender, and prescribed medication use in CP remains poorly understood, limiting optimization, safety, and equity of care. We examined how sex and gender are associated with repeated prescription refills of pain medications among individuals with CP.

**Methods:**

This study was conducted among individuals living with CP and links self-reported data to public and private prescription claims (*n* = 561). Repeated prescription refills of medications prescribed for CP and related comorbidities in the year following questionnaire completion were analyzed (≥40% days covered; yes/no; sensitivity analyses were performed using alternative cut-offs). Main independent variables were sex, gender identity, and gender-stereotyped personality traits (Bem Sex-Role Inventory). Cluster analysis was used to create intersecting sociodemographic subgroups (incorporating sex, gender, and other sociodemographic factors). Multivariable logistic regression was achieved to examine associations between these subgroups and repeated prescription refills.

**Results:**

Most commonly used medications prescribed for CP and related comorbidities were antidepressants (48%), anticonvulsants (35%), opioids (19%), and nonsteroidal anti-inflammatory drugs (18%) (repeated refills). Between clusters, statistically significant differences were found for the subgroups labelled: (1) ‘W*omen with private drug insurance*’, who had lower odds of repeated opioid prescription refills (_a_OR:0.38; 95%CI:0.15–0.95), and (2) ‘*Unemployed older men*’, who had lower odds of repeated antidepressant prescription refills (_a_OR: 0.45; 95%CI:0.24–0.87) (vs. ‘*Unemployed women*’).

**Conclusion:**

Our results highlight how sex, gender, and intersecting sociodemographic factors are associated with repeated prescription refills, particularly opioids and antidepressants, among individuals living with CP.

## Introduction

Chronic pain, commonly defined as pain lasting longer than three months (1), significantly impacts physical functioning, emotional well-being, and quality of life (2). Chronic pain requires a multimodal treatment approach that combines pharmacological, physical, and psychological interventions (3), but management remains suboptimal despite decades of clinical and research efforts (4). Affecting up to one in four individuals worldwide (5–8), chronic pain disproportionately affects women, gender-diverse individuals, and other equity-deserving populations (2), underscoring the need for an equity-oriented approach to care (9). In fact, many organizations and authors emphasize the urgent need for more equitable and effective treatments for all individuals living with chronic pain (9–13).

Pharmacological treatments remain an important component of chronic pain management and should be considered after weighing potential benefits and risks for each patient (14). Commonly used medication classes include nonsteroidal anti-inflammatory drugs (NSAIDs), anticonvulsants, antidepressants, acetaminophen, topical anesthetics, opioids, and prescribed cannabinoids (14, 15). In the real-world clinical context, 62% to 94% of individuals living with chronic pain use medications for chronic pain management (16–19). Yet pharmacological treatment poses many challenges: medications rarely reduce pain intensity to the desired level (20), they often need to be combined (e.g., analgesics and co-analgesics), which contributes to polypharmacy (21), and medications are associated with a wide range of side effects (18, 22).

To better understand the obstacles associated with pharmacological treatment in the real world and to address them more effectively, it is relevant to examine the biopsychosocial factors related to medication use (23). Sex [set of biological attributes associated with physical and physiological features including chromosomes, gene expression, hormone levels and function, and reproductive/sexual anatomy (24)] is a factor of particular interest in terms of chronic pain and its treatment (10). Evidence suggests differences in medication use between females and males: females are more likely to use acetaminophen and antidepressants (25), whereas males tend to use more NSAIDs (26) and strong opioids (27). However, it remains unclear whether these observations reflect true biological differences or the influence of gender [socially constructed roles, behaviors, expressions and identities of girls, women, boys, men, and gender diverse people (24)]. Without a clearer understanding of how sex and gender influence medication use, it is difficult to know which modifiable factors should be addressed to better support prescribing and use. To our knowledge, no study has simultaneously examined the associations of sex, gender, and other intersecting sociodemographic factors with medication use for chronic pain, as previous research has often considered sex alone. As highlighted in the Canadian Institutes of Health Research-Institute of Gender and Health's Action Plan (11), moving beyond simply describing sex or gender differences to investigating underlying factors, mechanisms, and their intersection is essential for a comprehensive understanding of health (11, 28, 29), including medication use.

The objective of this study was thus to examine the associations between sex, gender, and the use of medications prescribed for chronic pain and related comorbidities among individuals living with chronic pain. We conducted a sex- and gender-based analysis plus (SGBA+) accounting for other sociodemographic factors, including region of residence, country of birth, education level, employment status, and age. Drawing on an intersectional health perspective (30), we hypothesize that sex, gender, and these sociodemographic factors, particularly education, would influence medication use.

## Methods

This cohort study is reported in accordance with the Sex and Gender Equity in Research (SAGER) (31, 32) and the STROBE (33) guidelines checklists.

### Data sources

This study utilized data from the ChrOnic Pain trEatment (COPE) Cohort (34), a research platform designed to examine treatment utilization patterns, including medication, among individuals living with chronic pain. The dataset merges three sources: (1) a cross-sectional, web-based questionnaire administered between June and October 2019 to 1,935 individuals living with chronic pain; (2) longitudinal health administrative databases from the province of Quebec (Canada), which include information on healthcare utilization and medications dispensed in community pharmacies for patients covered by the universal public health insurance system (public prescription claims); and (3) data from reMed, which includes participants covered by private drug insurances (private prescription claims). To be eligible for the COPE Cohort, participants had to live with chronic pain [pain that persists or recurs for more than 3 months (1)], be at least 18 years old, reside in the province of Quebec (Canada), and be able to complete an online questionnaire in French. In Quebec, French is the sole official language and 93.7% of the population self-reports speaking French well enough to hold a conversation (35). Participants in the COPE Cohort (and those who consented to data linkage) were comparable to random (representative) samples of Canadians living with chronic pain in terms of age, employment status, education level, pain duration, pain intensity, and pain location (34). However, women were overrepresented in our sample (84% vs. 55%–65% in random samples of individuals living with chronic pain), highlighting the need to stratify prevalence results by sex and gender to assess the impact of this over-representation and to adjust accordingly.

To provide an appropriate context for an international audience, it is important to clarify how gender is conceptualized in the study population. The province of Quebec presents a distinct cultural and linguistic context, with significant transformations over the past four decades (36, 37) in both gender identities [how individuals perceive and define themselves (38)] and gender roles [socially expected behaviors and norms assigned to males and females that shape daily experiences and expectations (38)]. In recent years, there has been growing recognition and validation of diverse gender identities within Quebec society (39). At the same time, broader societal changes have fostered greater empowerment of women and increasing acknowledgment of men's engagement in traditionally feminine domains, such as caregiving and domestic responsibilities (40). This is why it is important to consider more than one gender construct at a time in order to fully understand the phenomenon.

As for health administrative databases used in the present study, the entire population is covered by a provincial universal health insurance program administered by the *Régie de l’assurance maladie du Québec* (RAMQ) (41). The health insurance covers the cost of outpatient medical visits, emergency department visits, hospitalizations and procedures offered to all residents (8.5 million people) (42). For prescription drugs, only a portion of the population is publicly covered: (1) people who are not eligible for private drug insurance with their employer or their partner's employer; (2) who are ≥65 years old; or (3) receiving last-resort financial assistance (41). These groups represent approximately 46% of the population (42) and 68% of the chronic pain population (18). The pharmaceutical services database (prescription claims) contains detailed information on the dispensing of prescribed drugs covered by the public plan, and the validity of its contents has been demonstrated (43). Since individuals covered by public insurance may represent a population that is older or of lower socioeconomic status (44), private insurance prescription claims were also harnessed as a source of data for medication use. Participants who reported being covered by a private prescription drug insurance were invited to register with the reMed registry (45). Established in 2009, this registry includes variables such as drug names, dose, formulation, quantity dispensed, fill dates, number of days of supply, number of authorized refills, dosage regimen, scrambled pharmacy identification number, scrambled prescriber identification number, and prescriber specialty for people covered by various private drug insurance plans (45).

### Study sample

**Figure 1:**
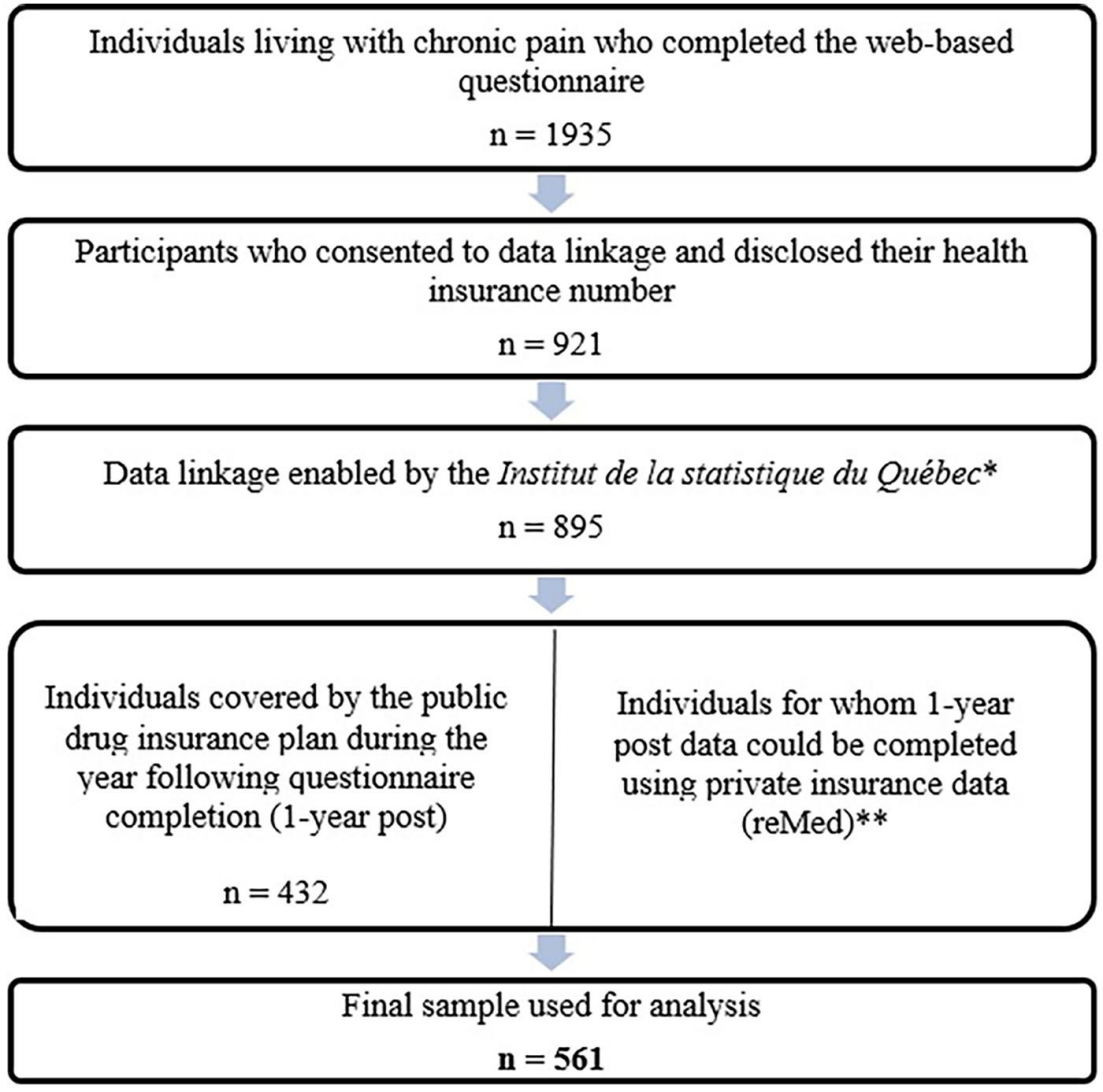
Data linkage flowchart leading to the study sample. * Data linkage was not possible for some participants due to inaccurate or missing identifying information (health insurance number, date of birth, first name, last name, sex). ** A total of 177 individuals who reported having private drug insurance consented to data linkage. For 48 of these participants, data from the public drug insurance database were sufficient to derive the study's medication-related variables, for example, in cases where a participant had private coverage at some point in the year prior to questionnaire completion but was covered by the public plan for the entire year following completion. Consequently, medication use variables were imported into the analytical dataset for the 129 participants for whom reMed data were available and for whom information on post-questionnaire (1-year) medication use was otherwise missing.

This study included participants from the COPE Cohort who provided their health insurance numbers in the web-based questionnaire and electronically consented to data linkage. Prescription claims were successfully matched by the *Institut de la Statistique du Québec* (ISQ) (Figure 1). Our study sample included 561 individuals for which complete public (*n* = 432) or private (*n* = 129) prescription claims were available for the entire year following the completion of the web-based questionnaire.

### Ethics

The project was approved by relevant authorities: (1) *Université du Québec en Abitibi-Témiscamingue* (#2018-05-Lacasse, A.), (2) *Commission d’accès à l’information du Québec* (#1027251-S), and (3) *Institut de la statistique du Québec*. All COPE Cohort participants provided free and informed consent.

### Study variables

#### Prescribed medication use

Main classes of analgesics and co-analgesics commonly used in the management of chronic pain and its comorbidities (e.g., mood, sleep) were considered (15) and identified using public and private prescription claims. Medications were classified using international nonproprietary names, along with the American Hospital Formulary Service (AHFS) codes (46) for public prescription claims, and the Anatomical Therapeutic Chemical (ATC) (47) classification system for private prescription claims. Included prescribed drug classes consisted of (1) NSAIDs (e.g., celecoxib, ketoprofen); (2) topical NSAIDs (e.g., diclofenac sodium); (3) acetaminophen combined with an opioid (e.g., acetaminophen with codeine, with tramadol or with oxycodone); (4) opioids (e.g., fentanyl, oxycodone, hydromorphone, morphine); (5) partial opioid agonists (e.g., buprenorphine); (6) anticonvulsants (e.g., pregabalin, gabapentin); (7) antidepressants (e.g., amitriptyline, duloxetine, citalopram); (8) prescribed skeletal muscle relaxants (e.g., cyclobenzaprine); (9) prescribed cannabinoids (e.g., nabilone); and (10) antimigraine agents (e.g., triptans). In the province of Quebec (Canada), drugs that are typically available over the counter (e.g., some NSAIDs, single-ingredient acetaminophen) can sometimes appear in prescription claims if they are prescribed to take advantage of coverage, for a specific dosage, or in a controlled clinical context. It should be noted that the specific indications for which the medications were prescribed (e.g., antidepressant prescribed for pain or depression) were not verified in our study, as diagnostic codes identifying chronic pain cases are not valid in health administrative databases (48).

For each class of medication, utilization was assessed during the year following the date of questionnaire completion. We defined the index date as the date of questionnaire completion to ensure that key self-reported determinants measured in the questionnaire (e.g., sex, gender, pain characteristics) preceded medication use outcomes, allowing for temporal assessment from a potential explanatory perspective. Since a medication may have been used only once or for a few days (e.g., an opioid for acute pain), the analysis of “repeated prescription refills” was the focus of our analysis and is particularly relevant in the context of pain that persists or recurs for more than 3 months (chronic pain) (1). Regarding the threshold used to define repeated prescription refills, some studies in the pain field have applied a cut-off of 40% of days covered (153, 154), whereas adherence studies more commonly use a threshold of 80% (51–53). In the context of our study, we aimed to capture prescription refills rather than strict adherence, without assuming that the medication was continuously prescribed throughout the entire year. For this reason, repeated prescription refills was thus defined as having medication coverage for ≥40% of the year, based on the proportion of days covered (consecutive or non-consecutive days) (49, 50). To ensure the robustness of our findings, sensitivity analyses were conducted using alternative cut-off values (30%, 50%, 80%).

Any use, defined as at least one dispensation during the year following the date of questionnaire completion, was also assessed in prescription claims for reference.

#### Sex at birth

Sex at birth at the time of questionnaire completion was obtained from health administrative records (*Régie de l’assurance maladie du Québec*), where male and female categories were available. This variable is considered reliable, as residents of Quebec have only been able to request a change to their sex designation on birth certificates since 2022. The provincial government further introduced the non-binary option on health insurance cards and driver's licenses as of 2024 (54, 55). Therefore, these policy changes do not impact on our study, which is based on data from 2020 and earlier.

#### Gender identity

Gender identity reflects how individuals understand and define themselves, whether as men, women, non-binary, gender fluid, two-spirit, and other identities (38). This concept differs from sex assigned at birth and can offer insight into psychosocial elements that may influence treatment utilization (56). In Canada, approximately 100,800 individuals (0.33%) identify as transgender and non-binary people in 2025, with Quebec being the province with the second-highest number (57). In the COPE questionnaire, participants indicated their gender by selecting one of the following options: woman, man, indeterminate, or unknown. This question was adapted from the National Institutes of Health (NIH) Task Force on Research Standards for Chronic Low Back Pain's minimal data set published in 2014 (58) and translated into Canadian French in 2017 (59). For our analysis, the gender diverse category included those who selected ‘indeterminate’ and ‘unknown’ answers or whose gender response differed from the sex at birth (transgender individuals).

#### Gender-stereotyped personality traits

In the COPE Cohort, gender-stereotyped personality traits were assessed using a validated French adaptation of the Bem Sex-Role Inventory (BSRI) consisting of 18 items (60, 61). The BSRI is a validated tool originally comprising 60 items (60), with several shorter versions available, including the one employed in this study (61). This French adaptation was chosen because it is brief and considered appropriate for individuals with different literacy levels (items are understood by adolescents) (61). Each item was scored on a 7-point Likert scale (1 = never true; 7 = always true) (60). Items (10 items for the feminine subscale and 8 for the masculine subscale) were averaged to obtain a feminine subscale score (0 to 70) and a masculine subscale score (0 to 56) (61). These 2 scores can be used as continuous variables, or combined for the creation of four subgroups using a median split approach (62, 63): (1) stereotypically feminine traits; (2) stereotypically masculine traits; (3) androgynous traits; and (4) undifferentiated traits. Specifically, those categorized as ‘feminine’ tend to describe themselves as tender and sensitive to others; those categorized as ‘masculine’ describe themselves as athletic, with leadership and self-confidence; those categorized as ‘androgynous’ score high on all these traits, and ‘undifferentiated’ individuals score low on all traits. Thus, classification should be interpreted accordingly. In our COPE Cohort sample, the internal consistency and factor structure of Fontayne et al.'s short version of the BSRI were found to be adequate (18), i.e., Cronbach's alphas of 0.90 [95% confidence interval (95% CI) = 0.89–0.91] and 0.82 (95% CI = 0.81–0.84) were obtained for the feminine and masculine scales respectively; confirmatory factor analysis reproduced the five first-order factors (tenderness, sensitivity to others, athletic, leadership, self-confidence) and two second-order factors (feminine, masculine) of the theoretical model published by Fontayne et al. (2000) with acceptable goodness of fit indices; *χ*^2^ (125) = 1,202.62 *p* < 0.0001, GFI = 0.9008, CFI=0.9147, RMSEA=0.0823) (18). Although criticized by some authors [e.g., femininity and masculinity vary across cultures and eras (64)], the BSRI remains the most widely used validated instrument for assessing traits beyond gender identity and has been validated in various contexts (65).

#### Covariables

In addition to sex and gender-related variables, other sociodemographic characteristics measured in the questionnaire were: Age (continuous), country of birth (Canada vs. outside of Canada), indigenous and race self-identification [Statistics Canada's Canadian Community Health Survey questions (66)], employment status (unemployed, part time, full time), highest level of education completed [secondary diploma or below, postsecondary diploma (below university level), university diploma)], and region of residence [six of the seventeen regions of the province are qualified as remote resource regions by the Quebec government (66) which allowed such a classification]. Self-reported pain characteristics included: Pain duration (classified as over 3 months but less than a year, 1–4 years, 5–9 years and ≥ 10 years), pain intensity [0–10 numerical rating scale measuring the average pain intensity in the last 7 days, classified as mild, moderate and severe (67)], various pain locations as dichotomous variables (yes/no), multisite pain (defined as ≥2 sites), presence of generalized pain, and pain frequency (continuous or occasional). Catastrophic thinking was assessed by agreeing/disagreeing with the following statement “*I feel that my pain is terrible and it's never going to get any better*”. This single item from the Pain Catastrophizing Scale (68) is referred to as “catastrophizing” in the National Institute of Health minimal dataset for chronic low back pain (58) and in the STarT Back Screening Tool (69). Neuropathic pain was evaluated using the validated DN4 Interview part (a score of ≥3/7 indicates a likely presence of neuropathic pain) (70), and pain interference using the Brief Pain Inventory (BPI) Interference Scale (71), which ranges from 0 to 10. Self-reported treatment and health status variables included: Pharmacological pain treatment use (current use of over-the-counter medication yes/no, current use of prescribed medication yes/no), perceived general health [one item from SF-12 (72)], percentages of pain relief [participants reaching substantial pain relief, i.e., 50% (73)], excessive polypharmacy [defined as the concurrent use of ≥10 medications for pain or other health problems (74)], side effects associated with pain medications, use of cannabis for pain management, physical and/or psychological pain treatments use (combined in one category as chronic pain is a disease of biopsychosocial origin and treatments can have an impact on both physical and psychological components at the same time), and access to a trusted healthcare professional for pain management. The questionnaire also inquired about feeling the need to reduce alcohol or drug consumption (never, rarely, sometimes, often consumed more than wanted), smoking habits (smokers, not a smoker, smoked in the past), and psychological distress using the validated Patient Health Questionnaire 4-item (PHQ-4) (75).

Variables determined using health administrative data (1-year time window before questionnaire completion) included public/private prescription drug insurance status, all cause healthcare contacts (visits to a family physician, to other medical specialists, emergency visits, hospitalizations), and a comorbidity score [combined Charlson (76) & Elixhauser (77) index with the International Classification of Diseases 9th revision]. This score evaluates the presence and severity of multiple health conditions, with higher scores indicating greater risk of complications and mortality (78, 79).

### Statistical analysis

#### Descriptive statistics

Means, standard deviations, counts, and percentages, were used to summarize the study population characteristics. As only three participants identified as gender-diverse, they were included in the overall study population description, but it was impossible to form a statistically sound subgroup for all subsequent analyses. Proportions were calculated to determine the prevalence of use of the ten medication classes under study during the year following the index date, both in terms of repeated prescription refills (medication coverage for ≥40% of the year) and any use (at least one prescription claim in the year).

#### Sex-and gender-based analysis plus

A sex and gender-based analysis plus (SGBA+) was conducted according to recommendations (28, 80). SGBA is an approach that systematically examines sex-based (biological) and gender-based (sociocultural) differences between men, women, boys, girls, and gender-diverse people (24, 38). We focused on repeated prescription refills as the dependent variable and examined the most commonly used drug classes (four stood out in the descriptive analysis, each used by roughly one in five or more individuals: antidepressants, anticonvulsants, opioids and prescribed NSAIDs). Repeated prescription refills were first compared across sex at birth (female, male), gender identity (women, men, gender-diverse), and gender-stereotyped personality trait (feminine, masculine, androgynous, undifferentiated) subgroups using bivariable tests (Chi-square tests and Tukey-style multiple comparisons of proportions tests).

Sex and gender are not independent of other characteristics such as age, race, and education, as they can interact with each other and with other characteristics to influence health outcomes, as indicated by the “plus” factor of SGBA+ (81). Such sociodemographic factors are important to understand the impact of sex and gender on medication use, and consider individuals holistically (29). In settings where various social determinants of health intersect to produce disparities, and where incorporating numerous interactions within a multivariable model proves difficult, considering alternative statistical methods that incorporate intersectional factors can be beneficial (28, 82, 83). We thus aimed to compare repeated prescription refills across intersecting sociodemographic subgroups. Such a group-based approach is recommended among strategies to incorporate intersecting variables into statistical analysis (82). Cluster analysis was chosen over other cross-sectional group-based methods (e.g., latent class analysis) as it does not assume independence of variables, and is simple to implement (84). Our approach is an innovative person-centered analysis that identifies subgroups based on common traits and interrelated theoretical dimensions (85), and was used in previous SGBA+ studies (84, 86).

A two-step cluster analysis was used, which allows integration of categorical and continuous variables (87, 88), and incorporated the following intersecting sociodemographic variables: categorical variables included sex at birth, gender identity, residence in a remote region, country of birth, education level, employment, and insurance status; continuous variables included age and gender-stereotyped personality traits (feminine and masculine scores; the continuous score was used rather than the categorical variable to enhance the precision of the cluster analyses). These sociodemographic variables were selected *a priori* among available COPE Cohort variables. All intersecting factors related to sex and gender that were available in the cohort were included following recommendations from the Canadian Institutes of Health Research – Institute of Gender and Health (11). The optimal number of intersecting sociodemographic subgroups was determined based on theory [important variables associated with sex and gender (11, 89)] and statistical validity, that is: (1) the Bayesian Information Criterion (BIC) (90), confirmed via graphical assessment and the elbow method (91, 92); (2) the average silhouette coefficient (93), a measure of how similar individuals are to their cluster compared to other clusters (ranging from −1 to 1, where values from 0.50 to 1.0 indicate good fit, values between 0.30 and 0.50 indicate fair fit, and values from −1.0 to 0.30 indicate poor fit) (94); (3) the cluster size ratio (largest to smallest cluster), to verify we have an appropriate number of people in each cluster (95); we tested models with 2 to 8 clusters (see all our steps in Supplementary Content 1); and (4) adequate interpretability of intersecting sociodemographic subgroups (e.g., to be able to assign meaningful labels to each group by observing the variables for which the groups differ the most). Subgroup labels were intended to reflect predominant profile patterns. For example, although most individuals in the ‘*Unemployed older men*’ subgroup were unemployed, this subgroup may still include some employed participants. The intersecting sociodemographic subgroups resulting from the cluster analysis then served as the categorical independent variable in the logistic regression analysis investigating their association with repeated prescription refills.

#### Multivariable analysis

Multivariable logistic regression models were employed to examine the association between intersecting sociodemographic subgroups (independent variable: reference category was ‘*Unemployed women’ as it was the most informative*) and repeated prescription refills (1 model for each of the most commonly used medication classes: antidepressants, anticonvulsants, prescribed NSAIDs, and opioids), adjusting for potential confounders. Covariables were preselected *a priori* according to the latest recommendations for multivariable model construction (96). Selection was based on existing literature, clinical relevance, and the Andersen model (97), which identifies predisposing, enabling, and need factors related to healthcare or medication utilization (98, 99). Owing to our substantial sample size, this method was favored over criticized selection techniques such as relying on bivariate regression analysis *p*-values (96) or stepwise selection (100). Adjusted odds ratios (aOR) and 95% confidence intervals (CIs) were calculated. Multicollinearity problems were screened (all variance inflation factors were below 5) (101), and Hosmer-Lemeshow tests (*p* > 0.05) supported the goodness of fit of the models. Variables included in the final models were missing completely at random (MCAR), as indicated by the results of Little's MCAR test (Chi-square=2.545, DF = 4, *p* = 0.637). Listwise deletion was thus an acceptable approach for our analysis (100, 102). Sensitivity analyses were conducted to verify if changing the cut-off for repeated prescription refills to 30%, 50%, or 80% days covered modified our main conclusions. We also explored whether changing the reference category of our main independent variable (intersecting sociodemographic subgroups) revealed additional differences. Although our silhouette coefficient was considered fair, sensitivity analyses, by changing the reference group, were therefore essential to ensure the validity of the clusters. All analyses were performed using SPSS Statistics version 30® (IBM Corp, Armonk, NY) and SAS version 9.4® (SAS Institute, Cary, NC).

## Results

### Characteristics of our study sample

Among the 1,935 COPE Cohort participants, a total of 561 participants were included in the analysis (those for which the linkage was successful and for which prescription claims were available; Figure 1). The study sample characteristics are presented in Table 1. The mean age was 52.7 ± 13.8 years, with 80.9% female (*n* = 452) and 19.1% male (*n* = 107). In terms of gender identity, 80.2% identified as women, 19.0% as men, and 0.5% as gender-diverse individuals. Regarding gender-stereotyped personality traits, 24.1% reported feminine traits (tenderness, sensitivity), 18.2% masculine traits (leadership, athleticism, self-confidence), 30.8% androgynous traits (high scores on both masculine and feminine traits), and 26.9% undifferentiated traits (low scores for both masculine and feminine traits).

**Table 1:** Study sample characteristics.

Characteristics (*n* = 561)	No. (%) of participants^a^
Sex at birth	
Male	107 (19.1)
Female	452 (80.9)
Gender identity	
Men	106 (19.0)
Women	450 (80.5)
Gender-diverse	3 (0.5)
Gender-stereotyped personality traits (categorized BSRI scores)	
Feminine	118 (24.1)
Masculine	89 (18.2)
Androgynous	151 (30.8)
Undifferentiated	132 (26.9)
Age (years) – mean ± SD	52.7 ± 13.8/
median (IQR)	54.0 (42.0–64.0)
Country of birth	
Canada	526 (96.5)
Outside Canada^b^	19 (3.5)
Indigenous self-identification	
Non-Indigenous	525 (98.1)
Indigenous peoples	10 (1.9)
Race self-identification	
White	537 (99.1)
Racialized	5 (0.9)
Employment	
Unemployed	406 (74.5)
Worker part-time	53 (9.7)
Worker full time	86 (15.8)
Education level	
Secondary diploma or below	129 (23.8)
Post-secondary diploma (below university level)	197 (36.4)
University diploma	215 (39.8)
Region of residence	
Remote^c^	110 (20.0)
Non-remote	440 (80.0)
Pain duration (years)	
Over 3 months but less than a year	16 (2.9)
1–4	116 (20.7)
5–9	115 (20.5)
≥ 10	313 (55.9)
Pain intensity in the past 7 days (categorized 0–10 score)	
Mild (1–4)	157 (28.4)
Moderate (5–7)	305 (55.3)
Severe (8–10)	90 (16.3)
Four most common pain locations^d^	
Back	318 (56.7)
Neck	257 (45.8)
Shoulders	253 (45.1)
Legs	230 (41.0)
Self-reported current use of prescribed pain medications	
No	86 (15.6)
Yes	467 (84.4)
Self-reported current use of over-the counter pain medications	
No	211 (38.1)
Yes	343 (61.9)
Self-reported current use of physical/psychological pain treatments use	
No	98 (17.5)
Yes	457 (81.5)
Healthcare utilization (1 year time window before questionnaire completion) – mean ± SD/median (IQR)	
Visits to a family physician	4.2 ± 4.0/3.0 (1.0–6.0)
Visit to other medical specialists	1.3 ± 2.2/0.0 (0.0–2.0)
Emergency visit	0.7 ± 1.4/0.0 (0.0–1.0)
Hospitalization	0.2 ± 0.5/0.0 (0.0–0.0)

SD: standard deviation; BSRI: Bem sex-role inventory; IQR: interquartile range.

a Unless stated otherwise.

b Outside Canada: Algeria, Germany, Belgium, New England, Colombia, Cuba, the United States, France, Haiti, Italy, Laos, Lebanon, Morocco, Poland, Democratic Republic of the Congo, Senegal, Switzerland, Venezuela.

c Remote resource regions as defined by Revenu Quebec (i.e., the provincial revenue agency): Bas-Saint-Laurent, Saguenay—Lac-Saint-Jean, Abitibi-Témiscamingue, Côte-Nord, Nord-du-Québec, Gaspésie—Îles-de-la-Madeleine. Non-remote regions are near a major urban centre.

d Categories not mutually exclusive. The proportion of missing data across the presented variables ranges between 0 and 12.7%The proportion of missing data across the presented variable ranges between 0 and 12.7%.

### Medication use prevalence in the whole sample

**Figure 2:**
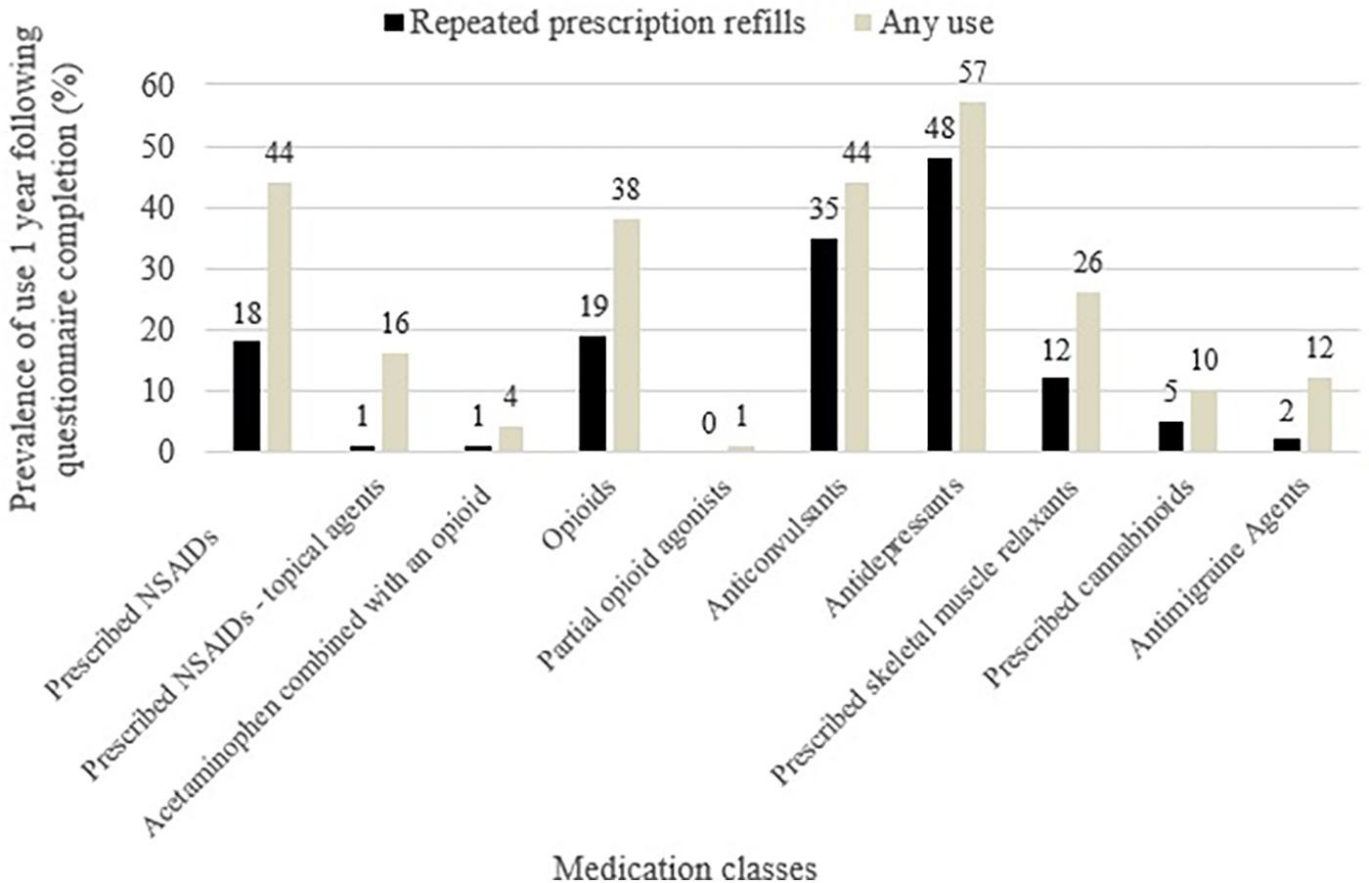
Prevalence of prescribed medication use 1 year post index date. Any use: at least one dispensation; Repeated prescription refills: ≥40% of days covered.

Figure 2 presents the prescribed classes of medication used for chronic pain and associated comorbidities (repeated prescription refills and any use), i.e., prescribed NSAIDs, prescribed NSAIDs - topical agents, acetaminophen combined with an opioid, opioids, partial opioid agonists, anticonvulsants, antidepressants, prescribed skeletal muscle relaxants, prescribed cannabinoids, antimigraine agents. Antidepressants (48%), anticonvulsants (35%), opioids (19%), and prescribed NSAIDs (18%) were the four most commonly used medications in terms of repeated prescription refills. The same four medication classes ranked highest for any use.

### Medication use across sex and gender subgroups

To investigate potential bivariable differences in repeated prescription refills by sex and gender, we focused on the four most commonly used medication classes (Table 2). Compared to females, males demonstrated a higher prevalence of repeated opioid prescription refills (26.2% vs. 17.0%, *p* = 0.030). The same result was observed for gender identity. Differences were also observed across gender-stereotyped personality traits subgroups for antidepressants (*p* = 0.005) and anticonvulsants (*p* = 0.014); with individuals exhibiting more feminine traits showing higher use. Given the observed sex- and gender-based differences in medication use, we then aimed to gain a more comprehensive understanding of individuals’ profiles through cluster analysis.

**Table 2:** Repeated prescription refills of the four most commonly used prescribed medications examined by sex and gender.

Repeated prescription refills (1 year post index date)	Antidepressants		Prescribed NSAIDs		Anticonvulsants		Opioids	
	*n* (%)	*p*-value	*n* (%)	*p*-value	*n* (%)	*p*-value	*n* (%)	*p*-value
Sex at birth								
Female (*n* = 452)	221 (48.9)	0.203	78 (17.3)	0.423	157 (34.7)	0.738	**77** **(17.0)**	**0**.**030**
Male (*n* = 107)	45 (42.1)		22 (20.6)		39 (36.4)		**28** (**26.2)**	
Gender identity								
Women (*n* = 450)	220 (48.9)	0.233*	78 (13.3)	0.549*	156 (34.7)	0.818*	**77** (**17.1)**	**0**.047*
Men (*n* = 106)	45 (42.4)		21 (19.8)		38 (35.8)		**27** (**25.5)**	
Gender-diverse (*n* = 3)	1 (33.3)		1 (33.3)		2 (66.7)		**1 (33.3)**	
Gender-stereotyped personality traits								
Feminine (*n* = 118)	**63** (**53.4)**	**0**.**005**	20 (16.9)	0.616	**54** (**45.8)**	**0**.**014**	28 (23.7)	0.296
Masculine (*n* = 89)	**41** (**46.1)**		11 (12.4)		**25** (**28.1)**		14 (15.7)	
Androgynous (*n* = 151)	**55** (**36.4)**		27 (17.9)		**44** (**29.1)**		23 (15.2)	
Undifferentiated (*n* = 132)	**74** (**56.1)**		25 (18.9)		**42** (**31.8)**		25 (18.9)	

*p*-values < 0.05 are reported in **bold.** The proportion of missing data across the presented variable ranges between 0.4% and 12.7%.

*Chi-square *p*-values may be invalid for comparisons across all three groups (women, men, gender-diverse) due to the small number in the gender-diverse group (*n* = 3). Therefore, we present *p*-values for comparisons between men and women only, to assess whether results based on sex and gender identity were comparable.

### Intersecting sociodemographic subgrouping via cluster analysis

**Figure 3:**
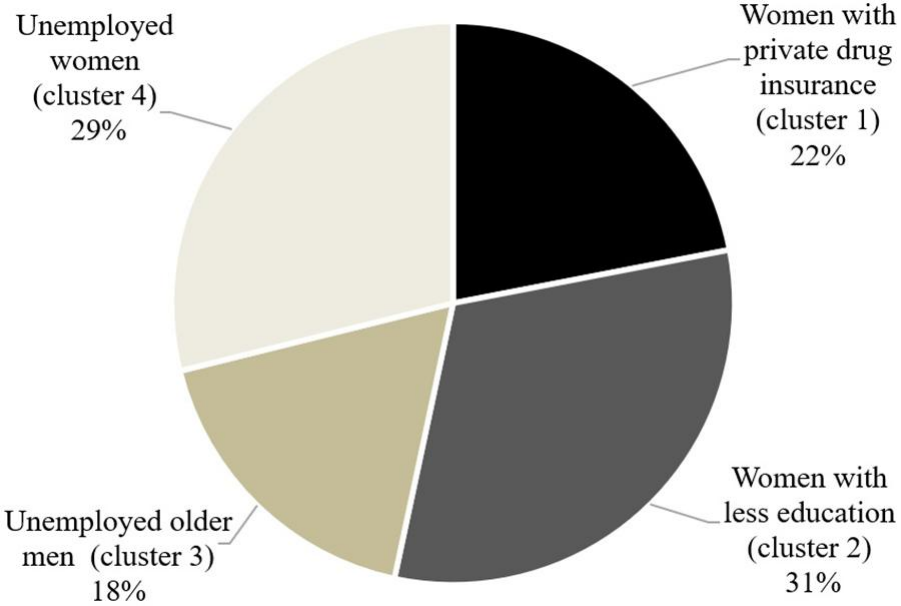
Intersecting sociodemographic subgroups with specific labels.

The elbow method indicated an optimal fit for a number of clusters between 2 and 8 (Supplementary Content 1**)**. As suggested in the literature (84, 103), we opted for a clustering solution that offers both an adequate statistical fit and yields interpretable clusters with clear qualitative distinctions. Based on these theoretical and validity criteria, a four-cluster solution emerged as the most suitable and providing the best overall fit (two clusters=poorer BIC, better silhouette coefficient, poorer cluster size ratio, poorer interpretability; three clusters=poorer BIC, same silhouette coefficient, better cluster size ratio, poorer interpretation; five clusters=better BIC, same silhouette coefficient, poorer cluster size ratio, poorer interpretation; six-seven-eight clusters: better BIC, better silhouette coefficient, poorer cluster size ratio, poorer interpretation). The silhouette coefficient of our four-cluster solution (SC = 0.3) was fair, and the cluster size ratio (1.76) was good (Supplementary Content 1). Each cluster represented more than 5% of the sample and at least 30 observations per group (104), and importantly, the clusters exhibited good interpretability. According to the cluster analysis, the three variables which had the greatest influence on the groups were: sex at birth, gender identity, and drug insurance status. Our classification labelling was as follows: cluster 1 ‘*Women with private drug insurance*’; cluster 2 ‘*Women with less education*’; cluster 3 ‘*Unemployed older men*’; and cluster 4 ‘*Unemployed women’* (Figure 3). Discriminant short labels were given to each subgroup, but the complete characteristics of each subgroup can be found in Table 3. As stated, discriminant short labels were identified based on characteristics that distinguished them from the other groups. For example, if all four groups were predominantly composed of individuals living in urban areas, or if three groups were predominantly composed of highly educated individuals, these characteristics were not included in the short label. The clusters were specifically designed to highlight characteristics unique to each group, in order to aid in the interpretation of the results. This did not mean that 100% of the group members matched the label.

**Table 3:** Descriptive profile of intersecting sociodemographic subgroups.

*n* = 478	Cluster 1 Women with private drug insurance *n* (%)	Cluster 2 Women with less education *n* (%)	Cluster 3 unemployed older men *n* (%)	Cluster 4 unemployed women *n* (%)
Cluster distribution	105 (22.0)	150 (31.4)	85 (17.8)	138 (28.9)
Sex at birth				
Female	105 (100)	150 (100)	0 (0.0)	138 (100)
Male	0 (0.0)	0 (0.0)	85 (100)	0 (0.0)
Gender identity				
Women	105 (100)	149 (99.3)	0 (0.0)	137 (99.3)
Men	0 (0.0)	0 (0.0)	84 (98.8)	0 (0.0)
Gender-diverse	0 (0.0)	1 (0.7)	1 (1.2)	1 (0.7)
Gender-stereotyped personality traits (BSRI continuous scores) – mean ± SD				
Feminine	5.8 ± 0.9	5.7 ± 0.9	5.3 ± 1.1	5.7 ± 0.9
Masculine	3.7 ± 1.1	3.7 ± 1.2	3.8 ± 1.2	3.8 ± 1.0
Age (years) – mean ± SD	46.7 ± 11.0	50.7 ± 14.3	58.2 ± 13.1	54.5 ± 14.1
Country of birth				
Canada	101 (96.2)	141 (94.0)	83 (97.6)	138 (100)
Outside Canada	4 (3.8)	9 (6.0)	2 (2.4)	0 (0.0)
Employment				
Worker full time	48 (45.7)	63 (42.0)	15 (17.6)	0 (0.0)
Unemployed	57 (54.3)	87 (58.0)	70 (82.4)	138 (100)
Education level				
Post-secondary diploma	93 (88.6)	77 (51.3)	64 (75.3)	138 (100)
No post-secondary diploma	12 (11.4)	73 (48.7)	21 (24.7)	0 (0.0)
Region of residence				
Remote	29 (27.6)	50 (33.3)	14 (16.5)	0 (0.0)
Urban	76 (72.4)	100 (66.6)	71 (83.5)	148 (100)
Drug insurance status				
Private	105 (100)	0 (0.0)	10 (11.8)	0 (0.0)
Public	0 (0.0)	150 (100)	75 (88.2)	138 (100)

### Repeated prescription refills across intersecting sociodemographic subgroups

**Figure 4:**
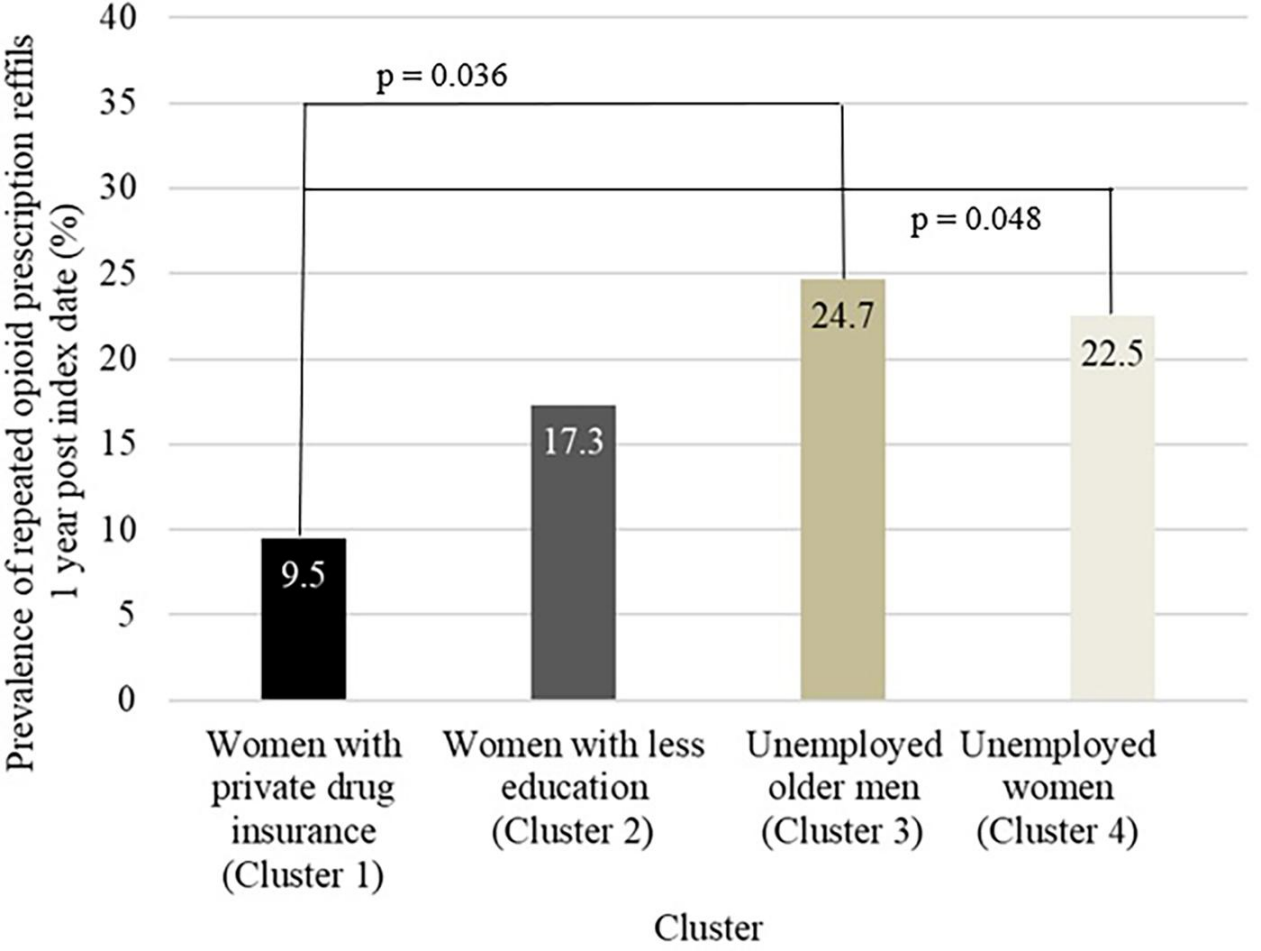
Proportion of repeated opioid prescription refills across intersecting sociodemographic subgroups. The line indicates a statistically significant difference (*p* < 0.05) between the two corresponding groups (*post hoc* Tukey-style multiple comparisons of proportions test).

Repeated prescription refills prevalences across intersectional subgroups are presented in Table 4. According to the bivariable analysis, only repeated opioid prescription refills appeared to differ between subgroups (*p* = 0.025) (Table 4). *Post hoc* Tukey-style multiple comparisons of proportions test revealed that ‘*Women with private drug insurance*’ were less likely to have repeated opioids prescription refills when compared to ‘*Unemployed older men*’ (9.5% vs. 24.7%, *p* = 0.036) or ‘*Unemployed women*’ (9.5% vs. 22.5%, *p* = 0.048) (Figure 4). The *p*-value was borderline for repeated antidepressants prescription refills (*p* = 0.057).

**Table 4:** Repeated prescription refills across intersecting sociodemographic subgroups.

Repeated prescription refills (1 year post index date) *n* = 478	Antidepressants		Prescribed NSAIDs		Anticonvulsants		Opioids	
	*n* (%)	*p*-value	*n* (%)	*p*-value	*n* (%)	*p*-value	*n* (%)	*p*-value
Women with private drug insurance (Cluster 1)	43 (41.0)	0.057^a^	11 (10.5)	0.221	27 (25.7)	0.222	**10 (9.5)**	**0.025**
Women with less education (Cluster 2)	78 (52.0)		30 (20.0)		55 (36.7)		**26 (17.3)**	
Unemployed older men (Cluster 3)	32 (37.6)		16 (18.8)		27 (31.8)		**21 (24.7)**	
Unemployed women (Cluster 4)	72 (52.2)		25 (18.1)		51 (37.0)		**31 (22.5)**	

*p*-values < 0.05 are reported in **bold**.

a Borderline to significance. The proportion of missing data across the presented variable is 14.8%.

The multivariable analysis revealed an association between intersecting sociodemographic subgroups and repeated opioid and antidepressants prescription refills independently from pain characteristics and health status; such an association was absent for prescribed NSAIDs or anticonvulsants. Table 5 presents the key findings of the opioid and antidepressant models. After adjusting for potential confounders, the models indicated that: (1) membership to the ‘*Women with private drug insurance’* subgroup, as compared to the ‘*Unemployed women*’ subgroup, significantly reduced the odds of repeated opioid prescription refills (aOR: 0.38; 95% CI: 0.15–0.95, *p* = 0.038); and (2) membership to the ‘*Unemployed older men*’ subgroup, as compared to the ‘*Unemployed women*’ subgroup, significantly reduced the odds of repeated antidepressant prescription refills (aOR: 0.45; 95%CI:0.24–0.87, *p* = 0.017).

**Table 5:** Multivariable model exploring associations between intersecting sociodemographic subgroups and repeated opioid and antidepressant prescription refills.

Variable	Repeated opioid prescription refills			Repeated antidepressant prescription refills		
	Adjusted Odds ratio (aOR)	*p*-value	95% CI	Adjusted Odds ratio (aOR)	*p*-value	95% CI
Intersecting sociodemographic subgroups ‘Unemployed women’ (reference)						
‘Women with private drug insurance’	**0**.**38**	**0**.**038**	**0.15–0.95**	0.81	0.494	0.44–1.49
‘Women with less education’	0.77	0.494	0.37–1.62	1.22	0.492	0.70–2.12
‘Unemployed older men’	0.84	0.677	0.38–1.87	**0**.**45**	**0**.**017**	**0.24–0.87**

Complete multivariable results are provided in Supplementary Content 3. *p*-values < 0.05 are reported in **bold**, 95% CI: 95% confidence interval and OR: odds ratio. The multivariable analysis was adjusted for the following covariables: pain duration, pain intensity in the last 7 days, multisite pain, generalized pain, pain frequency, agreeing with the statement ‘’I feel that my pain is terrible and it's never going to get any better’’, evidence of neuropathic pain according to the DN4 scale, pain interference according to the BPI score, excessive polypharmacy (≥10 medications), side effects associated with medication, use of cannabis for pain management, physical/psychological pain treatments use, access to a trusted health care professional for pain management, drug and alcohol use, smoking habits, psychological distress according to the PHQ scale, having prescription drug insurance, and comorbidity score (Charlson & Elixhauser index). In total, 444 participants (79.1%) were included in the final model (117 missing data; 20.9% were excluded).

Sensitivity analyses confirmed the stability of the above-mentioned conclusions when we changed the cut-off for defining repeated prescription refills to 30%, 50%, or 80%. In the repeated opioid prescription refills model, changing the reference category of our main independent variable (intersecting sociodemographic subgroups) did not reveal additional differences. In the repeated antidepressant prescription refills model, changing the reference category revealed an additional association: membership to the ‘*Women with less education*’ subgroup, as compared to the ‘*Unemployed older men*’ subgroup, significantly increased the odds of repeated antidepressant prescription refills (aOR: 2.69; 95%CI: 1.40–5.18, *p* = 0.003). Complete multivariable results (for the four main classes of pain medications studied) are provided in Supplementary Content 2 (including all other statistically significant covariates).

## Discussion

This study examined the associations between sex, gender, and other sociodemographic factors involved in the repeated prescription refills of prescribed pain medications among individuals living with chronic pain. Although various sex and gender differences are observed in the real world (as shown by our bivariable analysis of antidepressants, anticonvulsants and opioids use), the SGBA+ suggests that drug insurance status and employment are the drivers of such differences, independently of pain characteristics and health status. In the multivariable analysis, opioids and antidepressants were the classes of medications for which intersecting sociodemographic subgroups were a significant determinant. Specifically, women with private drug insurance were less likely to have repeated opioid prescription refills compared to other groups of women or men. Men were less likely to have repeated antidepressant prescription refills.

### Medication use prevalence in the whole sample

Our study stands out from previous work by focusing on repeated prescription refills, which we consider to be a more accurate reflection of the risks and benefits associated with chronic medication use. In contrast, many existing studies have estimated prevalence based on self-reported current use (cross-sectional design) and/or a single dispensation in prescription claims (27, 105, 106), approaches that may be biased because of medication use for acute pain. In our study, antidepressants, anticonvulsants, opioids, and prescribed NSAIDs were the medication classes with the greater prevalence of repeated prescription refills. Other Canadian and US studies of chronic pain populations similarly identified these medication classes as the most frequently used (107–109). The predominance of antidepressants and anticonvulsants use is also consistent with current national and international guidelines, which recommend prioritizing non-opioid medications for chronic pain management (110–113). Recent international data further support this trend, showing that among older adults, chronic pain has become the leading indication for antidepressant prescriptions, surpassing depression (114). Although prescribed NSAIDs were among the most frequently used medications in our cohort (18% with repeated prescription refills and 44% with at least one dispensation during the year), these proportions are lower than those reported in other Canadian studies [67%–73% (19, 27)], but higher than in one recent US study (109). This discrepancy is possibly due to differences in populations or data type and data capture, as our data includes only prescribed NSAIDs. It may also reflect variations in prescribing practices or differences in clinical guidelines. For anticonvulsants, they are primarily recommended for neuropathic pain (115), but interestingly, even within our general chronic pain population, their use appears to be relatively high (35% with repeated prescription refills and 44% with at least one dispensation in the year). Few data are available on the prevalence of anticonvulsant use among individuals living with chronic pain, which appears highly variable, ranging from 7% to 44% (19, 107, 109). It is worth noting that anticonvulsant (e.g., gabapentinoid) use has increased in recent years (116), and a significant share of this use is off-label (prescribed outside their approved indications, dosages, or target populations) (117). With regard to opioids, use in our cohort (19% with repeated prescription refills and 38% with at least one dispensation in the year) appears relatively comparable to what is typically reported in the literature, where proportions typically hover around 25% (108, 118). Opioids remain a highly debated class of analgesics due to concerns about addiction, tolerance, and adverse effects (119). This elevated percentage is preoccupying given that Canadian guidelines recommend optimizing non-opioid pharmacological and non-pharmacological therapies before considering opioid trials in patients with chronic pain (111). The high average number of years living with pain in our cohort, however, suggests that our participants are not new users and have likely tried several other medications beforehand.

It is worth noting that, in our cohort, certain treatments, such as opiate partial agonists, prescribed NSAIDs – topical agents, and acetaminophen in combination with opioids, were rarely or not used at all, with prevalence of repeated prescription refills ranging from 0% to 1%. This pattern is consistent with findings in the literature where partial opioid agonists also ranked among the least frequently used treatments for chronic pain (108), and where opioid-acetaminophen combinations are recommended to be used with caution in Canadian guidelines (120).

### Medication use across sex and gender subgroups

Our results were presented in a stepwise manner to follow a logical SGBA+ framework. First, in the bivariable analyses, sex- and gender-related differences were observed. Males/men, compared to females/women (sex/gender identity), showed higher prevalence of repeated opioid prescription refills, which was already reported in the literature (27, 121, 122). In addition, statistically significant differences between gender-stereotyped personality traits and the use of antidepressants and anticonvulsants (but not opioids) were identified; these findings have not been previously reported in the literature. Individuals exhibiting more feminine personality traits appeared to have higher use of antidepressants and anticonvulsants. Previous research has suggested that gender stereotypes, such as the perception that ‘women are more emotional than men’ (123), may lead healthcare providers to interpret women's physical symptoms as psychological in nature. This process, often referred to as the psychologizing of women's pain, could potentially help contextualize the disproportionate prescription of antidepressants to women (123). Multiple factors may also help explain the association, such as clinical presentation, comorbidities (e.g., anxiety), healthcare-seeking behaviors, prescribers’ behaviors, and expectations, making the identification of sex and gender differences at the bivariable level a starting point.

When differences are observed, a deeper understanding, i.e., one that includes other intersecting identity factors, is necessary, highlighting the importance of using an SGBA+ approach. In the literature, sex and/or gender differences in medication use remain inconsistent. Some studies report that men use more of certain medications, while others find higher use among women. This is true for opioids: some studies report greater use among men (27, 121, 122), whereas others show higher use among women (124). Medications like antidepressants and anticonvulsants are commonly associated with higher use among women (25, 121). What if sex or gender differences (consistently used interchangeably in the literature, further increasing the challenges) reflected deeper intersecting sociodemographic factors? Who are these men and women, and where can we intervene to optimize treatment use? In our study, cluster analysis and the inclusion of multiple intersecting variables were essential to uncover these patterns, as recommended in the literature (82). Identifying these underlying drivers is critical: if we aim to reduce disparities in treatment use, interventions should target key structural barriers; for instance, improving healthcare access for the uninsured, or addressing broader inequalities in employment and insurance coverage at the population level (82). Indeed, our findings confirmed that sex/gender differences exist, but only within specific subgroups. For example, in our study, women with private drug insurance were significantly less likely to have repeated opioid prescription refills compared to unemployed older men and unemployed women. In this case, gender identity, employment status, and insurance coverage all appear to influence medication use. Regarding anticonvulsants, once intersecting sociodemographic factors were considered, the initial gender-stereotyped personality traits differences disappeared. This highlights the “Plus” in SGBA+, suggesting that these apparent gender differences may actually reflect underlying structural disparities related to factors such as employment and insurance. Although gender bias in prescribing practices likely exists (125), other contributing factors must also be considered. After adjusting for potential confounders such as pain characteristics and health profile (e.g., pain duration, intensity and interference, multisite or generalized pain, neuropathic pain, polypharmacy, anxiety/depression, comorbidity score), no significant differences remained between males and females (sex at birth variable) in opioid use. This suggests that sex alone did not explain the use, but rather its intersection with other identity dimensions. A thorough mediation analysis would be a very interesting avenue for future research.

One plausible explanation for the lower opioid use among privately insured women may be their improved access to alternative treatments (126). Individual with private insurance are more likely to afford or access non-pharmacological options (126, 127) (e.g., physiotherapy, massage therapy, acupuncture, psychotherapy), which could reduce the need for opioid initiation and repeated prescription refills. Although we adjusted for the use of non-pharmacological treatments in the multivariable model, it is however possible that private insurance affords higher quality, more frequent, or more sustained non-pharmacological care than what is captured by the binary (“yes/no”) variable defining non-pharmacological treatments used in the model. Furthermore, private insurance is often linked to employment. Discussions with our patient partners led us to hypothesize that persons who are employed may avoid opioid use due to concerns about dependence, cognitive impairment, or stigma, especially in work environments that require high levels of concentration and performance. Supporting this, prior studies have shown that employed individuals generally use fewer opioids, though the mechanisms remain unclear (128, 129). In contrast, unemployed women may encounter greater barriers to accessing non-pharmacological care and may also face higher levels of social isolation, a known risk factor for opioid use (130, 131). Social support networks are known to help with pain and reduce reliance on medication; in their absence, individuals may turn more readily to pharmacological options.

Our finding of lower antidepressant use among men aligns with previous literature (25, 132, 133), and may reflect gender differences in help-seeking (134, 135), underdiagnosis of depression (136, 137), stigma (138–140), or lower adherence or persistence with prescribed treatment (141). In contrast to the psychologizing of women's pain mentioned earlier, these findings illustrate that gender norms can negatively affect both genders in different ways: while women may be prescribed more antidepressants, older unemployed men may be under-treated due to societal expectations of stoicism and reluctance to seek help. Further analyses could be conducted to explore these factors in more depth; unfortunately, they were not measured in our study.

### Recommendations

Our findings suggest that opioid and antidepressant use is not only associated to clinical needs but also to access to care, employment status, and social context. These are factors that deserve further investigation to inform more equitable, effective and safe pain management strategies. In future studies about medication use, applying a SGBA+ (including variables such as sex, gender, and other intersecting sociodemographic factors) is therefore essential to better understand these disparities and to inform more targeted and equitable recommendations. Individuals (women and men) who are unemployed and/or uninsured should have access to services, including non-pharmacological pain management options, which, although they may involve upfront costs, are generally cost-effective (142, 143). Specific support should be implemented for unemployed women to promote an integrative approach to care and, where appropriate, reduce opioid use.

For healthcare professionals, it is important to provide care that responds to everyone's specific needs and to actively assess patients’ socioeconomic resources and constraints. For example, developing targeted prevention programs that consider sociodemographic factors, such as employment, insurance status, and income, could particularly benefit people living in precarious conditions. However, this requires time with the patients, underscoring the potential importance of involving multiple healthcare professionals, particularly those who may not be constrained in time by a fee-for-service model, such as primary care nurses who can play a role in patient advocacy and longitudinal follow-up (144).

### Strengths and limitations

Our study presents several strengths, notably the inclusion of a large, diverse and province-wide sample of participants from Quebec, Canada. Even though the study was conducted in Quebec, it is possible to generalize our findings more broadly. While gender and sex contexts may vary, our sample included representation across all sexes and gendered personality traits. Frequency of membership to these subgroups may differ from one country to another, but the associations with outcomes measured using multivariable analysis are unlikely to change. Because of this, and due to the representativeness of our sample across multiple sociodemographic variables and pain characteristics, we are confident that the study demonstrates good generalizability to other provinces of Canada (similar healthcare systems and prescription medications available are the same). Self-reported data, considered the gold standard in pain research (145), was collected following best practices and established guidelines, using validated measurement tools (59, 146–149). The linkage between self-reported and longitudinal health administrative data helped to overcome challenges typically associated with identifying chronic pain cases based solely on administrative data (48). This linkage allowed for precise longitudinal information on prescription medication use, while also providing a wide array of sociodemographic and clinical variables related to pain. Prescription claims offer a key advantage: they enable longitudinal tracking of medication dispensing while avoiding the potential for recall bias, which is expected to be challenging in chronic pain populations considering the high frequency of polypharmacy (150). A known limitation of such databases is, however, that they record prescriptions filled, but do not confirm actual medication intake. However, this bias was likely reduced for some classes of medications, because we focused on repeated prescription refills (>40% of days covered in the year). In fact, individuals who consistently collect their medications from the pharmacy are less likely to purchase medications they do not intend to use since they pay a portion of the cost through the deductible and co-insurance (except for medications with resale potential, such as opioids). Keeping in mind that the threshold used to define repeated prescription refills is inherently arbitrary and may affect classification, sensitivity analyses demonstrated that the conclusions remained stable when varying the cut-off to 30%, 50%, or 80%. Notably, the triple data linkage with the reMed database represents a significant strength (45). It allowed us to include individuals with private drug insurance (almost never included in Canadian database studies), thereby enhancing the representativeness of this study. This is particularly important, as those covered by public drug insurance tend to be older and have lower socioeconomic status due to the eligibility criteria for public drug insurance (44).

One of the main limitations of this study is the small number of gender-diverse participants (*n* = 3) in our cohort, for which it was not possible to conduct inferential statistical analyses. At least, they could be included in most of our descriptive statistics. Targeted recruitment strategies could be implemented, and future studies should focus on gender-diverse populations (151, 152). Additionally, our intersectional profile did not include Indigenous or racial identity due to the small number of participants in these categories. This is a notable limitation, given the known health disparities faced by these populations (2). The non-inclusion of the race variable may have influenced the composition of the generated clusters; for example, the ‘*Unemployed women*’ or ‘*Women with less education*’ clusters might differentially represent racialized groups. Future research with larger and more diverse samples is needed to examine how intersecting factors, including race and ethnicity, shape patterns of medication use for chronic pain.

Finally, although the BSRI is a widely used and validated tool (65), it presents some limitations. Since societal attitudes, roles, and expectations related to gender have evolved considerably in recent years, the BSRI may not fully capture contemporary understandings of gender identity and expression (64). The development of a tool better suited to today's diversity represents an interesting avenue for future research. Finally, while we observed associations between intersectional profiles and repeated prescription refills, conducting a formal mediation analysis to examine the mechanisms underlying differences should be addressed in future research.

## Conclusion

When examining the associations between sex, gender, and repeated prescription refills, the consideration of additional intersecting sociodemographic factors is essential, making the application of SGBA+ a critical analytical tool. The findings that women with private drug insurance were less likely to have repeated opioid prescription refills as compared to unemployed women, and that unemployed older men were less likely to have repeated antidepressant prescription refills, highlight key socioeconomic, structural, and public health issues. Whether this difference in use is appropriate or not, to ensure equitable pain management, assessing both clinical and socioeconomic factors could help guide patients more effectively toward better and safer care. Our study showed that repeated opioid prescription refills remain frequent in real-world settings (1 out of 5) which warrants particular attention. As for other medications classes such as prescribed NSAIDs and anticonvulsants, sex and gender differences are real, but are explained by other sociodemographic factors.

## Data Availability

The datasets presented in this article are not readily available because the COPE Cohort participants did not initially provide consent to open data. Data are available from the corresponding author upon reasonable request and conditionally to a proper ethical approval for a secondary data analysis. As for health administrative databases, access must be granted by the Institut de la statistique du Québec (ISQ) (data holder). Programming codes can be obtained directly from the corresponding author. Requests to access the datasets should be directed to anais.lacasse@uqat.ca.
