# Supplementary material for: Sex, gender, and sociodemographic factors associated with repeated prescription refills in chronic pain: insights from a prescription claims cohort study
Source: Front Pain Res (Lausanne). 2026 Apr 22;7:1810867. doi: 10.3389/fpain.2026.1810867 (PMC13143939; doi:10.3389/fpain.2026.1810867)
Supplement: Supplementary file 1 [file Datasheet1.pdf]

SUPPLEMENTARY CONTENT 1

Autoclustering BIC values by number of clusters, silhouette coefficient, and cluster size ratio:

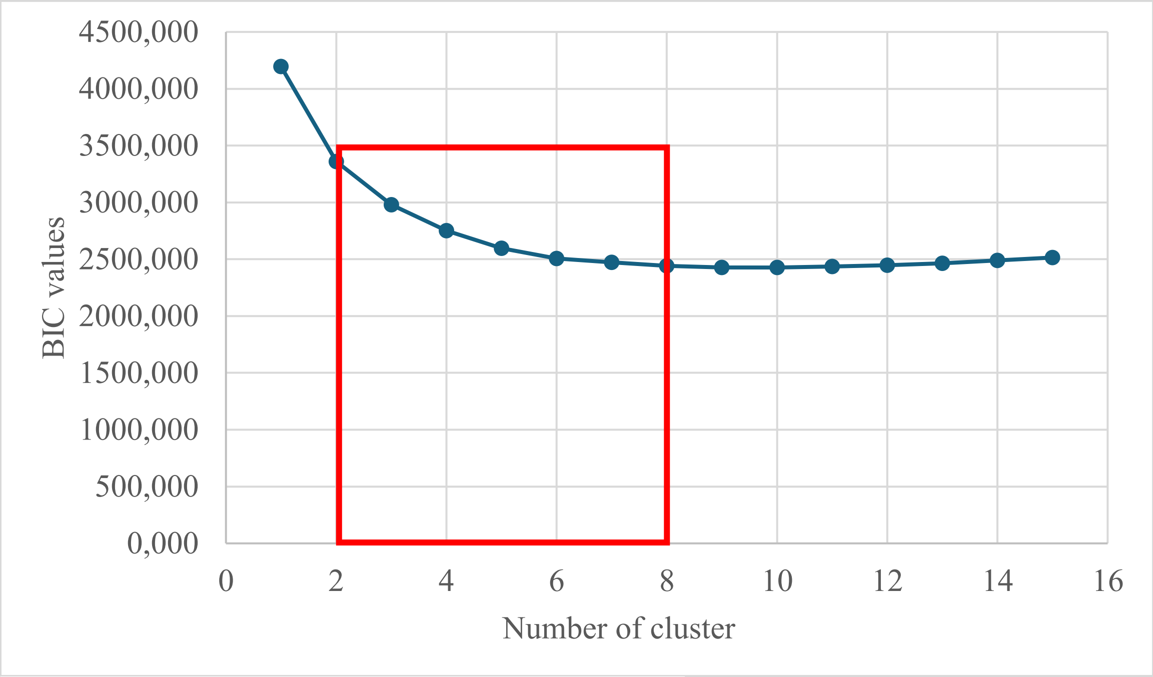

Récapitulatif du modèle

**Algorithme**	TwoStep
**Entrées**	10
**Clusters**	4

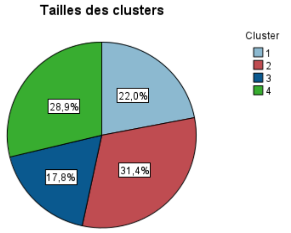

Qualité des clusters

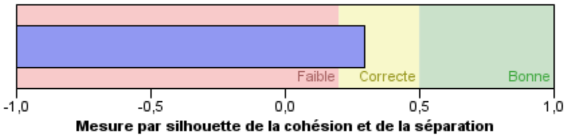

Taille du cluster le plus petit	85 (17,8%)
Taille du cluster le plus grand	150 (31,4%)
Rapport des tailles : Cluster le plus grand par rapport au cluster le plus petit	1,76

The following variables were used to realize the cluster analysis: age, sex at birth, gender identity, gender personality traits according to the BSRI, country of birth, employment, education level, region of residence, and drug insurance status * Outside Canada: Algeria, Germany, Belgium, New England, Colombia, Cuba, United States, France, Haiti, Italy, Laos, Lebanon, Morocco, Poland, Democratic Republic of Congo, Senegal, Switzerland, Venezuela. ** Remote resource regions as defined by Revenu Quebec (i.e., the provincial revenue agency): Bas-Saint-Laurent, Saguenay—Lac-Saint-Jean, Abitibi-Témiscamingue, Côte-Nord, Nord-du-Québec, Gaspésie—Îles-de-la-Madeleine. Non-remote regions are near a major urban center. BSRI: Bem Sex Role Inventory

SUPPLEMENTARY CONTENT 2

A. Integral results of the multivariable model exploring associations between subgroups and repeated **opioid** prescription refills .

Variable	Odds ratio (OR) – Adjusted β	p-value	95% CI
Intersecting sociodemographic subgroups (vs. 'unemployed women')			
'Women with private drug insurance'	**0.38**	**0.038**	**0.15 – 0.95**
'Women with less education'	0.77	0.494	0.37 – 1.62
'Unemployed older men'	0.84	0.677	0.38 – 1.87
Pain duration in year (continuous)	**1.04**	**<0.001**	**1.02 – 1.06**
Pain intensity in the last 7 days (vs. mild; score 1-4)			
Moderate (score 5-7)	1.29	0.529	0.58 – 2.87
Severe (score 8-10)	0.65	0.428	0.23 – 1.87
Multisite pain (≥2 sites) (yes vs. no)	0.64	0.483	0.19 – 2.22
Generalized pain (yes vs. no)	1.70	0.109	0.89 – 3.27
Pain frequency (occasionally vs. continuously)	0.24	0.079	0.05 – 1.18
Agreeing with the statement '' I feel that my pain is terrible and it's never going to get any better'' (yes vs. no)	0.99	0.974	0.48 – 2.05
Evidence of neuropathic pain according to the DN4 scale (yes vs. no)	1.04	0.902	0.57 – 1.88
Pain interference according to the BPI score (continuous)	1.14	0.193	0.94 – 1.38
Excessive polypharmacy (≥10 medications) (yes vs. no)	**2.91**	**<0.001**	**1.60 – 5.28**
Side effects associated with medications (yes vs. no)	**0.25**	**0.002**	**0.10 – 0.61**
Use of cannabis for pain management (yes vs. no)	0.67	0.206	0.35 – 1.25
Physical and/or psychological pain treatments use (yes vs. no)	**2.50**	**0.012**	**1.23 – 5.09**
Access to a trusted health care professional for pain management (yes vs. no)	1.16	0.693	0.56 – 2.37
Drug and alcohol use (Have you consumed alcohol or drugs more than you would have liked vs. never)			
Rarely	**0.43**	**0.042**	**0.19 – 0.97**
Sometimes	**0.16**	**0.002**	**0.05 – 0.52**
Often	1.06	0.918	0.37 – 3.02
Smoking habits (vs no smokers)			
Smokers	1.71	0.178	0.78 – 3.73
I smoked in the past, but not anymore	0.96	0.893	0.49 – 1.87
Anxiety-Depression symptoms – PHQ Score (vs. none; score 0-2)			
Mild (score 3-5)	1.05	0.915	0.45 – 2.43
Moderate (score 6-8)	1.22	0.680	0.48 – 3.12

Severe (score 9-12)	1.31	0.584	0.50 – 3.47
Having private prescription drug insurance (yes vs. no)	0.25	0.095	0.05 – 1.28
Comorbidity score (Charlson & Elixhauser index) (continuous)	1.36	0.076	0.97 – 1.92

Table footnotes: Complete multivariable results are provided in Supplementary Content 4. p-values < 0.05 are reported in **bold**, 95% CI: 95% confidence interval and OR: odds ratio. The multivariable analysis was adjusted for the following covariables: pain duration, pain intensity in the last 7 days, multisite pain, generalized pain, pain frequency, agreeing with the statement ''I feel that my pain is terrible and it's never going the get any better'', evidence of neuropathic pain according to the DN4 scale, pain interference according to the BPI score, excessive polypharmacy (≥10 medications), side effects associated with medication, use of cannabis for pain management, physical/psychological pain treatments use, access to a trusted health care professional for pain management, drug and alcohol use, smoking habits, psychological distress according to the PHQ scale, having private prescription drug insurance, and comorbidity score (Charlson & Elixhauser index). In total, 444 participants (79.1%) were included in the final model (117 missing data; 20.9% were excluded).

B. Integral results of the multivariable model exploring associations between subgroups and repeated **antidepressant** prescription refills.

**Variable**	**Odds ratio (OR) – Adjusted β**	**p-value**	**95% CI**
Intersecting sociodemographic subgroups (vs. 'unemployed women')<br>'Women with private drug insurance'<br>'Women with less education'<br>'Unemployed older men'	 <br>0.81<br>1.22<br>**0.45**	 <br>0.495<br>0.492<br>**0.017**	 <br>0.44 - 1.49<br>0.70 - 2.12<br>**0.24 - 0.87**
Pain duration in year (continuous)	1.00	0.759	0.99 - 1.02
Pain intensity in the last 7 days (vs. mild; score 1-4)<br>Moderate (score 5-7)<br>Severe (score 8-10)	 1.04<br>**0.38**	 0.900<br>**0.020**	 0.59 - 1.82<br>**0.17 - 0.86**
Multisite pain (≥2 sites) (yes vs. no)	0.67	0.307	0.31 - 1.44
Generalized pain (yes vs. no)	1.41	0.171	0.86 - 2.29
Pain frequency (occasionally vs. continuously)	0.50	0.073	0.23 - 1.07
Agreeing with the statement '' I feel that my pain is terrible and it's never going to get any better'' (yes vs. no)	0.82	0.449	0.48 - 1.38
Evidence of neuropathic pain according to the DN4 scale (yes vs. no)	**1.63**	**0.032**	**1.04 - 2.55**
Pain interference according to the BPI score (continuous)	0.94	0.409	0.82 - 1.10
Excessive polypharmacy (≥10 medications) (yes vs. no)	**3.32**	**<0.001**	**2.06 - 5.37**
Side effects associated with medications (yes vs. no)	**0.47**	**0.004**	**0.28 - 0.79**
Use of cannabis for pain management (yes vs. no)	0.86	0.543	0.54 - 1.38
Physical and/or psychological pain treatments use (yes vs. no)	**2.38**	**0.005**	**1.31 - 4.32**
Access to a trusted health care professional for pain management (yes vs. no)	1.29	0.346	0.76 - 2.18
Drug and alcohol use (Have you consumed alcohol or drugs more than you would have liked vs. never)<br>Rarely<br>Sometimes<br>Often	 <br>0.64<br>1.01<br>1.76	 <br>0.126<br>0.971<br>0.237	 <br>0.37 - 1.13<br>0.54 - 1.92<br>0.69 - 4.45
Smoking habits (vs no smokers)<br>Smokers<br>I smoked in the past, but not anymore	 1.62<br>1.08	 0.126<br>0.757	 0.87 - 3.00<br>0.67 - 1.73
Anxiety-Depression symptoms – PHQ Score (vs. none; score 0-2)<br>Mild (score 3-5)<br>Moderate (score 6-8)<br>Severe (score 9-12)	 <br>1.00<br>1.55<br>1.58	 <br>0.998<br>0.206<br>0.238	 <br>0.57 - 1.80<br>0.79 - 3.06<br>0.74 - 3.35

Having private prescription drug insurance (yes vs. no)	1.55	0.309	0.67 - 3.57
Comorbidity score (Charlson & Elixhauser index) (continuous)	0.81	0.190	0.59 - 1.11

Table footnotes: Complete multivariable results are provided in Supplementary Content 4. p-values < 0.05 are reported in **bold**, 95% CI: 95% confidence interval and OR: odds ratio. The multivariable analysis was adjusted for the following covariables: pain duration, pain intensity in the last 7 days, multisite pain, generalized pain, pain frequency, agreeing with the statement ''I feel that my pain is terrible and it's never going the get any better'', evidence of neuropathic pain according to the DN4 scale, pain interference according to the BPI score, excessive polypharmacy (≥10 medications), side effects associated with medication, use of cannabis for pain management, physical/psychological pain treatments use, access to a trusted health care professional for pain management, drug and alcohol use, smoking habits, psychological distress according to the PHQ scale, having private prescription drug insurance, and comorbidity score (Charlson & Elixhauser index). In total, 444 participants (79.1%) were included in the final model (117 missing data; 20.9% were excluded).

C. Integral results of the multivariable model exploring associations between subgroups and repeated **NSAIDs** prescription refills.

**Variable**	**Odds ratio (OR) – Adjusted β**	**p-value**	**95% CI**
Intersecting sociodemographic subgroups (vs. 'unemployed women')<br>'Women with private drug insurance'<br>'Women with less education'<br>'Unemployed older men'	 <br>0.58<br>0.10<br>1.06	 <br>0.230<br>0.992<br>0.890	 <br>0.24-1.41<br>0.47-2.10<br>0.46-2.42
Pain duration in year (continuous)	0.99	0.448	0.97 - 1.02
Pain intensity in the last 7 days (vs. mild; score 1-4)<br>Moderate (score 5-7)<br>Severe (score 8-10)	 **2.77**<br>1.16	 **0.015**<br>0.788	 **1.22 - 6.31**<br>0.39 - 3.48
Multisite pain (≥2 sites) (yes vs. no)	1.38	0.535	0.50-3.84
Generalized pain (yes vs. no)	0.78	0.455	0.41-1.50
Pain frequency (occasionally vs. continuously)	0.92	0.899	0.26 - 3.26
Agreeing with the statement '' I feel that my pain is terrible and it's never going to get any better'' (yes vs. no)	1.46	0.278	0.74 - 2.91
Evidence of neuropathic pain according to the DN4 scale (yes vs. no)	1.11	0.736	0.61 - 2.01
Pain interference according to the BPI score (continuous)	1.07	0.490	0.88 - 1.30
Excessive polypharmacy (≥10 medications) (yes vs. no)	**3.11**	**<0.001**	**1.70 - 5.70**
Side effects associated with medications (yes vs. no)	0.57	0.126	0.28 - 1.17
Use of cannabis for pain management (yes vs. no)	1.15	0.664	0.61- 2.20
Physical and/or psychological pain treatments use (yes vs. no)	**2.85**	**0.003**	**1.42 - 5.73**
Access to a trusted health care professional for pain management (yes vs. no)	1.37	0.400	0.66 - 2.85
Drug and alcohol use (Have you consumed alcohol or drugs more than you would have liked vs. never)<br>Rarely<br>Sometimes<br>Often	 <br>0.45<br>1.45<br>0.41	 <br>0.073<br>0.357<br>0.197	 <br>0.19 - 1.08<br>0.66 - 3.20<br>0.10 - 1.59
Smoking habits (vs no smokers)<br>Smokers<br>I smoked in the past, but not anymore	 **3.05**<br>1.45	 **0.005**<br>0.287	 **1.40 - 6.66**<br>0.73 - 2.84
Anxiety-Depression symptoms – PHQ Score (vs. none; score 0-2)<br>Mild (score 3-5)<br>Moderate (score 6-8)<br>Severe (score 9-12)	 <br>0.76<br>0.58<br>0.41	 <br>0.482<br>0.244<br>0.073	 <br>0.35-1.65<br>0.23-1.45<br>0.15-1.09

Having private prescription drug insurance (yes vs. no)	**0.11**	**0.041**	**0.01 - 0.91**
Comorbidity score (Charlson & Elixhauser index) (continuous)	1.04	0.801	0.76-1.43

Table footnotes: Complete multivariable results are provided in Supplementary Content 4. p-values < 0.05 are reported in **bold**, 95% CI: 95% confidence interval and OR: odds ratio. The multivariable analysis was adjusted for the following covariables: pain duration, pain intensity in the last 7 days, multisite pain, generalized pain, pain frequency, agreeing with the statement ‘’I feel that my pain is terrible and it’s never going the get any better’’, evidence of neuropathic pain according to the DN4 scale, pain interference according to the BPI score, excessive polypharmacy (≥10 medications), side effects associated with medication, use of cannabis for pain management, physical/psychological pain treatments use, access to a trusted health care professional for pain management, drug and alcohol use, smoking habits, psychological distress according to the PHQ scale, having private prescription drug insurance, and comorbidity score (Charlson & Elixhauser index). In total, 444 participants (79.1%) were included in the final model (117 missing data; 20.9% were excluded).

D. Integral results of the multivariable model exploring associations between subgroups and repeated **anticonvulsants** prescription refills.

**Variable**	**Odds ratio (OR) – Adjusted β**	**p-value**	**95% CI**
Intersecting sociodemographic subgroups (vs. 'unemployed women')			
'Women with private drug insurance'	0.71	0.321	0.37 - 1.39
'Women with less education'	1.22	0.510	0.68 - 2.18
'Unemployed older men'	0.90	0.775	0.46 - 1.80
Pain duration in year (continuous)	1.00	0.982	0.98 - 1.02
Pain intensity in the last 7 days (vs. mild; score 1-4)			
Moderate (score 5-7)	1.08	0.806	0.59 - 1.96
Severe (score 8-10)	0.77	0.540	0.34 - 1.76
Multisite pain (≥2 sites) (yes vs. no)	0.75	0.509	0.32 - 1.76
Generalized pain (yes vs. no)	1.09	0.743	0.65 - 1.81
Pain frequency (occasionally vs. continuously)	0.92	0.842	0.40 - 2.13
Agreeing with the statement '' I feel that my pain is terrible and it's never going to get any better'' (yes vs. no)	1.04	0.896	0.60 - 1.80
Evidence of neuropathic pain according to the DN4 scale (yes vs. no)	0.69	0.120	0.43 - 1.10
Pain interference according to the BPI score (continuous)	0.99	0.852	0.85 - 1.14
Excessive polypharmacy (≥10 medications) (yes vs. no)	**4.61**	**<0.001**	**2.84 - 7.50**
Side effects associated with medications (yes vs. no)	**0.41**	**0.003**	**0.23 - 0.73**
Use of cannabis for pain management (yes vs. no)	1.26	0.373	0.76 - 2.09
Physical and/or psychological pain treatments use (yes vs. no)	1.72	0.074	0.95 - 3.13
Access to a trusted health care professional for pain management (yes vs. no)	1.18	0.560	0.67 - 2.10
Drug and alcohol use (Have you consumed alcohol or drugs more than you would have liked vs. never)			
Rarely	0.80	0.457	0.44 - 1.45
Sometimes	1.16	0.667	0.60 - 2.25
Often	0.51	0.207	0.17 - 1.46
Smoking habits (vs no smokers)			
Smokers	**1.98**	**0.034**	**1.05 - 3.73**
I smoked in the past, but not anymore	0.77	0.323	0.46 - 1.29
Anxiety-Depression symptoms – PHQ Score (vs. none; score 0-2)			
Mild (score 3-5)	0.97	0.925	0.52 - 1.80
Moderate (score 6-8)	0.81	0.563	0.39 - 1.66
Severe (score 9-12)	0.59	0.180	0.27 - 1.28

Having private prescription drug insurance (yes vs. no)	1.24	0.621	0.53 - 2.94
Comorbidity score (Charlson & Elixhauser index) (continuous)	1.01	0.952	0.74 - 1.38

Table footnotes: Complete multivariable results are provided in Supplementary Content 4. p-values < 0.05 are reported in **bold**, 95% CI: 95% confidence interval and OR: odds ratio. The multivariable analysis was adjusted for the following covariables: pain duration, pain intensity in the last 7 days, multisite pain, generalized pain, pain frequency, agreeing with the statement ''I feel that my pain is terrible and it's never going the get any better'', evidence of neuropathic pain according to the DN4 scale, pain interference according to the BPI score, excessive polypharmacy (≥10 medications), side effects associated with medication, use of cannabis for pain management, physical/psychological pain treatments use, access to a trusted health care professional for pain management, drug and alcohol use, smoking habits, psychological distress according to the PHQ scale, having private prescription drug insurance, and comorbidity score (Charlson & Elixhauser index). In total, 444 participants (79.1%) were included in the final model (117 missing data; 20.9% were excluded).
